# FADD phosphorylation is critical for cell cycle regulation in breast cancer cells

**DOI:** 10.1038/sj.bjc.6602955

**Published:** 2006-01-31

**Authors:** S Matsuyoshi, K Shimada, M Nakamura, E Ishida, N Konishi

**Affiliations:** 1 Department of Pathology, Nara Medical University School of Medicine, Nara 634-8521, Japan

**Keywords:** Fas-associated death domain, c-jun N-terminal kinase, phosphorylation, cell cycle arrest, breast cancer, immunohistochemistry

## Abstract

Anti-oestrogen therapy is effective for control of hormone receptor-positive breast cancers, although the detailed molecular mechanisms, including signal transduction, remain unclear. We demonstrated here that long-term tamoxifen treatment causes G2/M cell cycle arrest through c-jun N-terminal kinase (JNK) activation, which is dependent on phosphorylation of Fas-associated death domain-containing protein (FADD) at 194 serine in an oestrogen (ER) receptor-positive breast cancer cell line, MCF-7. Expression of a dominant negative mutant form of MKK7, a kinase upstream of JNK, or mutant FADD (S194A) in MCF-7 cells suppressed the cytotoxicity of long-term tamoxifen treatment. Of great interest, similar signallings could be evoked by paclitaxel, even in an ER-negative cell line, MDA-MB-231. In addition, immunohistochemical analysis using human breast cancer specimens showed a close correlation between phosphorylated JNK and FADD expression, both being significantly reduced in cases with metastatic potential. We conclude that JNK-mediated phosphorylation of FADD plays an important role in the negative regulation of cell growth and metastasis, independent of the ER status of a breast cancer, so that JNK/FADD signals might be promising targets for cancer therapy.

Various therapeutic strategies have been clinically applied for patients with breast cancers, and the oestrogen receptor (ER) antagonist, tamoxifen, is a major drug with documented efficacy. The mechanisms have been suggested to involve cytotoxicity, and although the details remain unclear, signalling pathways appear to be essential.

c-jun N-terminal kinase (JNK) is a member of the serine/threonine family of protein kinases that is induced by various cellular stress agents like ultraviolet (UV), *γ* irradiation and cytotoxic drugs. c-jun N-terminal kinase activation plays an important role in induction of cell cycle arrest or cell death, and ionising or UV radiation has been found to cause G2/M cell cycle arrest through JNK phosphorylation in breast cancer cell lines (Mingo-Sion *et al*, 2004). Cell cycle arrest in the G2/M phase is mainly involved with cytotoxicity due to paclitaxel or synergistic effects with chemotherapy or ionising radiation (Tishler *et al*, 1992; Geard *et al*, 1993; Gagandeep *et al*, 1999).

The Fas-associated death domain-containing protein (FADD) was originally identified as an adapter molecule inducing a death-inducing signalling complex required for Fas-mediated apoptosis (Chinnaiyan *et al*, 1995; Nagata and Golstein, 1995). Recently, we have shown that FADD phosphorylation at Ser194 can be induced by paclitaxel, with impact on functions both upstream and downstream of the MEKK1/MKK7/JNK1 pathway, closely associated with sensitisation to chemotherapy in prostate cancer cells (Shimada *et al*, 2002, 2004). Involvement of FADD phosphorylation in cell cycle arrest has also been indicated in breast epithelial or tumour cells (Alappat *et al*, 2003).

In the present study, we investigated whether the JNK and FADD phosphorylation signals contribute to tamoxifen-induced cell growth arrest in ER-positive breast cancer cells, then tried to execute the signals and effect tumour suppression by using the JNK/FADD activator, paclitaxel, in ER-negative cells. In addition, the expressions of phosphorylated JNK/FADD were examined using human breast cancer specimens by immunohistochemistry and evaluated with reference to clinicopathological parameters.

## MATERIALS AND METHODS

### Cell culture and chemicals

The human breast cancer cell lines, MCF-7 (ER positive, p53-wt) and MDA-MB-231 (ER negative, p53-mt), were purchased from the American Type Culture Collection (Manassas, VA, USA) and cultured in RPMI supplemented with 5% fetal bovine serum. For establishment of long-term tamoxifen treatment, MCF-7 cells were exposed to low doses (0.01–1 *μ*M) of tamoxifen (Wako, Osaka, Japan) for 4 months, as detailed previously (Paulsen *et al*, 1996; Madsen *et al*, 1997). Anti-p53, anti-JNK1, anti-p38, anti-p21, anti-Bcl-2 and anti-actin antibodies were purchased from Santa Cruz Biotechnology (Santa Cruz, CA, USA) and a specific inhibitor of JNK (SP600125) from Calbiochem (San Diego, CA, USA). Paclitaxel and anti-FLAG antibodies were from Sigma-Aldrich Japan, Ltd (Tokyo, Japan) and anti-c-Myc antibodies were from Clontech, Tokyo, Japan. Phospho-FADD (Ser194), Phospho-SAPK/JNK (Thr183/tyr185) and Phospho-Bcl-2 (Ser70) antibodies were from Cell Signaling Technology (Beverly, MA, USA) and the anti-FADD antibodies were from Transduction Laboratories (Lexington, KY, USA). Antibodies to ER (M7047), progesterone receptor (M3569) and c-erbB-2 receptor (A0485) were from Dako (Dako Corp, Carpenteria, CA, USA).

### Tumour samples

A total of 107 cancer samples were obtained at surgical resection from patients in Nissei Hospital, Osaka and Nara City Hospital, Nara, Japan between 1987 and 2002. Neither chemotherapy nor radiation therapy was performed before surgery in any of the cases. Histopathological diagnoses were made using the WHO histological classification of tumours of the breast. Specimens were fixed in 10% buffered formalin, embedded in paraffin, sectioned and stained with haematoxylin–eosin (HE). Informed consent was obtained from patients before the collection of specimens as appropriate. Clinicopathological characterisics of 107 patients are shown in Table 1.

### Western blotting analysis

Cells were washed once with phosphate-buffered saline (PBS) and suspended in lysis buffer (40 mM Hepes (pH 7.4) with 10% glycerol, 1% Triton X-100, 0.5% Nonidet P-40, 150 mM NaCl, 50 mM NaF, 20 mM *β*-glycerol phosphate, 1 mM EDTA, 1 mM phenylmethylsulphonyl fluoride and 0.1 mM vanadate) with an added protease inhibitor mixture (1 mg ml^−1^ aprotinin, leupeptin and pepstatin). Cell lysates were resolved on sodium dodecyl sulphate (SDS)–polyacrylamide gels and transferred to polyvinylidene difluoride membranes (Millipore Ltd, Bedford, MA, USA). The membranes were blocked in Tris-buffered saline-Tween 20 (TBST) buffer (20 mM Tris-HCl (pH 7.5) containing 150 mM NaCl and 0.1% Tween 20) with 5% skim milk at room temperature for 1 h, then incubated with the indicated primary antibodies overnight at 4°C, washed with TBST, and incubated with anti-rabbit or anti-mouse IgG (Amersham Pharmacia Biotech, Tokyo, Japan) as the secondary antibody for 2 h. After washing with TBST, blots were detected on X-ray films using an enhanced chemiluminescence detection system.

### Flow cytometry and cell cycle analysis

After stimulation, adherent cells from one 35 mm dish were harvested by trypsinisation, washed in ice-cold PBS and fixed with 80% ethanol. After resuspension in PBS containing 50 mg ml^−1^ propidium iodide, 0.1% Nonidet P-40 and 100 mg ml^−1^ Rnase A (Sigma, Tokyo, Japan), and incubation for 1 h, the cell cycle distribution was analysed using a flow cytometry and Cellquest software (Becton Dickinson, San Jose, CA, USA).

### Preparation of constructs and stable clones, and transfection of expression vectors

The FLAG-tagged human FADD cDNA, prepared by conventional RT–PCR, was cloned into the mammalian expression vector pME18S. The following primers were used for preparing the resulting mutant of FADD, serine (S) 194 → alanine (A) (dephosphorylated FADD): 5′-GGAGTGGGGCCATGGCCCCGATGTCATGGAAC-3′ (Imai *et al*, 1999; Shimada *et al*, 2002). A plasmid Myc-tagged dominant negative MKK7 (MKK7 d/n), the mutagenic oligonucleotide (CAGGCCACATCATTGCTGTTCTGCAGATGCGGCGCTCTGGGAAC) was used to convert Lys165 of MKK7 to Leu (Moriguchi *et al*, 1997; Shimada *et al*, 2004). The mutation was confirmed by DNA sequencing. Fas-associated death domain-containing proteins S194A and MKK7 d/n were generated using a Quick-change Site-directed Mutagenesis kit (Stratagene, La Jolla, CA, USA). An expression vector for the Hygromycin-resistant gene (pTK-Hyg) was obtained from Clontech. Cells were seeded at 5 × 10^5^ cells well^−1^ in six-well plates, cultured in fresh medium for 24 h and then cotransfected with the pTK-Hyg vector harbouring the Hygromycin B (HygB)-resistant gene or the expression vectors (Invitrogen, Tokyo, Japan). Resistant colonies were isolated after 6 weeks by selection with HygB, then examined by Western blotting using antibodies.

### Transfection with nonsense or antisense p53

MCF-7 cells treated with long-term tamoxifen were seeded at 5 × 10^5^ cells well^−1^ in six-well plates, and transfected with a nonsense p53 oligonucleotide (NSO) (5′-GGAGCCAGGGGGGAGCAGGG-3′) or an antisense p53 oligonucleotide (ASO) (5′-CCCTGCTCCCCCCTGGCTCC-3′) (from Biomol Res. Lab., PA, USA) using LipofectAMINE (Invitrogen) according to the manufacturer's protocol. After 48 h, the cells were stimulated with the indicated reagents and the expression of p53 was analysed by Western blotting using anti-p53 antibodies (Shimada *et al*, 2003).

### Cell viability assay

Cell survivals were analysed by CellTiter 96TM nonradioactive cell proliferation assay (Promega, Madison, WI, USA). Briefly, 15 *μ*l of MTT reagent was added to each well followed by incubation at 37°C for 4 h to allow the formation of purple colour crystals of formazan. Then, 100 *μ*l of Solubilisation/Stop solution was added to each well, and the reaction mixture was incubated in the dark for 1 h at room temperature. The developed colour density was measured spectrophotometrically at 570 nm using the microplate reader. Assessment was performed in triplicate.

### Matrigel invasion assay

*In vitro* invasion assays were performed using Matrigel-coated wells (11 *μ*g per filter; 8 *μ*m, pore size). Briefly, 8 × 10^4^ cells of the control clone (HygB) and the stable clone overexpressing FADD S194A were placed in the insert. After 24 h incubation at 37°C, the chambers were scrubbed with a cotton bud to remove noninvading cells, and invading cells were fixed and stained with haematoxylin, then counted under a light microscope. The experiment was repeated three times.

### Immunohistochemical staining

Tumour sections 4 *μ*m thick were deparaffinised, and endogenous peroxidase was blocked by immersing in a 3% hydrogen peroxidase in methanol for 20 min. The slides were then immersed in 10 mM citrate buffer solution (pH 6.0) and placed in a pressure cooker for 10 min at 120°C. After cooling, immunohistochemistry was performed by the avidin–biotin complex technique using a histofine SAB-PO (R) Kit (Nichirei, Tokyo, Japan). Slides were rinsed with PBS and nonspecific binding was blocked with 10% goat normal serum (Histofine kit). Incubation with primary Phospho-FADD (Ser194) antibody (diluted 1 : 50; Cell Signaling Technology, Beverly, MA, USA) and Phospho-SAPK/JNK (Thr183/tyr185) antibody (diluted 1 : 50; Cell Signaling Technology, Beverly, MA, USA) was at 4°C overnight. The reaction products were visualised by immersing the slides in diaminobenzidine tetrahydrochloride (DAB) and finally counterstained with Mayer's haematoxylin. Phosphorylated JNK (P-JNK) and phosphorylated FADD (P-FADD) immunostainings were calculated as the percentage of positive primary cancer cells in relation to the total number in at least 10 representative fields. Nuclear stainings greater than 10% were judged as positive in oestrogen and progesterone receptor. Only membrane staining intensity and patterns were evaluated and the results were judged using Hercep Test Kit scoring guidelines in c-erbB-2 receptor (Table 1).

### Statistical analysis

Statistical analyses were performed using StatView 5.0 software (SAS Institute. Inc.). The Student *t*-test was used to detect differences between the expressions of P-JNK and P-FADD, positive and negative in lymph node metastasis. *Post hoc* test (Tukey–Kramer) and Spearman's correlation test were carried out to analyse the relations between P-JNK and P-FADD expressions. *P-*values less than 0.05 were considered to be statistically significant. All *P-*values were two sided.

## RESULTS

### Long-term tamoxifen treatment induces phosphorylation of JNK and G2/M cell cycle arrest in ER-positive breast cancer cells

**Figure 1:**
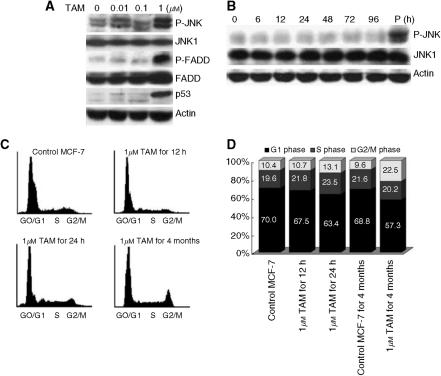
Long-term tamoxifen treatment and cell cycle arrest. (**A**) MCF-7 cells were grown with 5% fetal calf serum for 4 months in the presence or absence of 0.01–1 mM tamoxifen. Phosphorylated and nonphosphorylated forms of FADD and JNK, and p53 expression were assessed by Western blotting. (**B**) MCF-7 cells were stimulated with 1 *μ*M tamoxifen for the indicated times. Phosphorylated and nonphosphorylated forms of JNK expression were assessed by Western blotting. The positive control (P) was long-term 1 *μ*M tamoxifen-treated MCF-7 cells. (**C**) Cell cycle analyses of MCF-7 cells treated with 1 *μ*M tamoxifen for short (12 and 24 h) and long (4 months) periods were performed by flow cytometry. (**D**) Each proportions of cells in the G1, S and G2/M phase was calculated. Control MCF-7 for 4 M (fourth bar) was the cells cultured in the medium without tamoxifen for 4 months. P-JNK: phosphorylated JNK, P-FADD: phosphorylated FADD, TAM: tamoxifen.

As shown in Figure 1A, JNK was ubiquitously phosphorylated in MCF-7 cells grown in medium containing tamoxifen at concentrations of 0.01–1 *μ*M for 4 months, but the level was slight. The activity was little changed with the stimulation at 0.01–0.1 *μ*M tamoxifen, but it was significantly elevated with 1 *μ*M. With higher doses of tamoxifen, p53 protein and P-FADD expressions were increased, whereas p21 and phosphorylation of Bcl-2, p44/42 MAP kinase or p38 were not induced (data not shown). Short-term (6–96 h) stimulation with 1 *μ*M tamoxifen did not affect JNK phosphorylation (Figure 1B). Figure 1C and D shows changes in cell cycle distributions by tamoxifen for each incubation period. With short-term treatment (12 or 24 h), cell cycle distributions were not significantly affected, but the long-term treatment (4 months) caused G2/M arrest.

**Figure 2:**
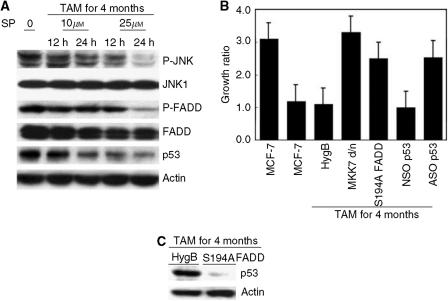
Fas-associated death domain-containing protein phosphorylation and p53 stabilisation are dependent on JNK activation. (**A**) MCF-7 cells grown in medium containing 1 *μ*M tamoxifen for 4 months were treated with the indicated concentrations of SP600125 for 12 or 24 h. Then, phosphorylated and nonphosphorylated forms of FADD and JNK, and p53 expression were assessed by Western blotting. (**B**) Stable clones expressing various mutant genes (control clone, HygB; kinase inactive mutant MKK7 clone, MKK7 d/n; S194A mutant FADD clone, S194A FADD) were generated and maintained in MCF-7 cells treated for 4 months at 1 *μ*M tamoxifen. Then, cell survivals for 72 h in medium in the presence of tamoxifen were analysed by MTT assay. MCF-7 cells incubated with tamoxifen for 4 months were transfected with NSO p53 or ASO p53. After 24 h incubation, cell survivals for 72 h were analysed. The representative data shown are for growth ratios compared to the cases before stimulation. (**C**) Control (HygB) and S194A mutant FADD clones were established as for (**B**), and p53 protein expression was assessed by Western blotting.

Recently, phosphorylation of JNK or FADD at 194 serine has been shown to be closely associated with cell cycle arrest in various types of cells (Scaffidi *et al*, 2000; Alappat *et al*, 2003; Mingo-Sion *et al*, 2004; Shimada *et al*, 2004). Therefore, we investigated the contribution of JNK and FADD phosphorylation and cell growth suppression induced by long-term tamoxifen in MCF-7 cells. As shown in Figure 2A, treatment with the specific JNK inhibitor, SP600125, for 12 or 24 h, reduced phosphorylation of JNK in a dose-dependent manner, and similarly inhibited phosphorylation of FADD at 194 serine. Moreover, p53 induction was also suppressed by JNK inhibition. Cell proliferation was strongly reduced by long-term tamoxifen treatment, but inhibition of JNK by transfection with a dominant negative mutant form of the upstream kinase of JNK, MKK7, or inhibition of FADD phosphorylation by overexpression of S194A mutant FADD or knock down of p53 by antisense oligonucleotide transfection restored almost normal cell growth activity to the cells (Figure 2B). Consistently, the long-term tamoxifen-induced cell cycle arrest at G2/M was also inhibited by the same treatments (data not shown). Interestingly, p53 induction by tamoxifen was cancelled by S194A mutant FADD overexpression (Figure 2C). When FADD was knocked down in MCF-7 cells with long-term tamoxifen treatment, p53 induction was strongly cancelled (data not shown). These results clearly demonstrate that JNK activation-dependent FADD phosphorylation is an upstream signal to stabilise p53, and that JNK/FADD/p53 signals are essential for growth arrest by long-term tamoxifen treatment in human breast cancer cells.

### Paclitaxel causes cytotoxic signals similar to tamoxifen in ER-negative breast cancer cells

**Figure 3:**
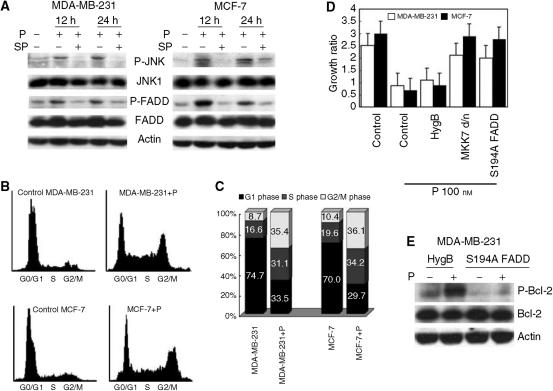
Paclitaxel induces cell cycle arrest through the JNK/FADD pathway in the human breast cancer cell lines, MDA-MB-231 and MCF-7. (**A**) Both cell lines were pretreated with or without 25 *μ*M SP600125 for 12 h, then stimulated with 100 nM paclitaxel for the indicated times. Phosphorylated and nonphosphorylated forms of JNK and FADD were assessed by Western blotting. Expression of actin was used as an internal control. (**B, C**) Cell cycle analyses in both cell lines treated with or without 100nM paclitaxel for 12 h were performed by flow cytometry as described in the ‘Materials and Methods’. (**D**) We constructed stable clones expressing the hygromycin-resistant gene (HygB, control), the kinase inactive mutant MKK7 (MKK7 d/n) or with S194A mutant FADD (S194A FADD) as described in the ‘Materials and Methods’. The parental line, MDA-MB-231 or MCF-7, and the stable clones were stimulated with 100 nM paclitaxel for 72 h, and cell survivals were analysed by MTT assay. The representative data are growth ratios compared to the cells before stimulation. (**E**) Control (HygB) and S194A mutant FADD clones were established as (**D**) in MDA-MB-231 cells, and were treated with or without 100 nM paclitaxel. Phosphorylated and nonphosphorylated Bcl-2 protein expressions were assessed by Western blotting. SP: SP600125, P: paclitaxel.

The cytotoxic signals induced by tamoxifen were not detected in an ER-negative breast cancer cell line, MDA-MB-231 (data not shown). Since we previously demonstrated activation of JNK and phosphorylation of FADD by paclitaxel in prostate cancer cells (Shimada *et al*, 2004), we examined the cytotoxicity of paclitaxel for long-term tamoxifen treatment, in MDA-MB-231. Paclitaxel at 100 nM phosphorylated JNK and FADD at 194 serine, and the FADD phosphorylation was inhibited by the JNK inhibitor, SP600125 (Figure 3A, left panels). In addition, paclitaxel induced cell cycle arrest at G2/M (Figure 3B, upper panels) (Figure 3C, left bars), and strongly suppressed cancer cell proliferation (Figure 3D, white bars) as assessed by both flow cytometry and MTT assay. Cell growth suppression in the presence of paclitaxel was significantly cancelled by inhibition of JNK or FADD phosphorylation when dominant negative mutant MKK7 (MKK7 d/n) or S194A mutant FADD were overexpressed (Figure 3D, white bars). Phosphorylation of Bcl-2 plays an important role in cell cycle arrest compensatory for p53, which was also observed in MDA-MB-231 treated with paclitaxel (data not shown). Moreover, the Bcl-2 phosphorylation by paclitaxel was inhibited when S194A mutant FADD was overexpressed in MDA-MB-231 (Figure 3E). The same mechanisms appeared also to be operating in ER-positive MCF-7 breast cancer cells: paclitaxel at the same concentration caused JNK activation/FADD phosphorylation, and cell cycle arrest at G2/M, which were cancelled by the JNK inhibitor or S194A mutant FADD overexpression (Figure 3A, right panels) (Figure 3B, lower panels) (Figure 3C, right bars) (Figure 3D black bars).

### Dephosphorylated FADD accelerates cancer cell invasion and metastatic potential

**Figure 4:**
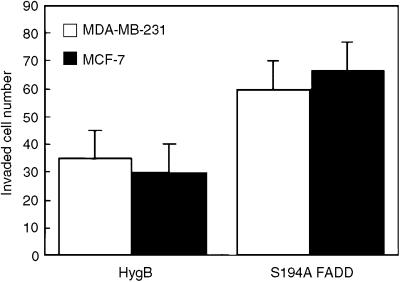
Dephosphorylated FADD enhances cell invasive ability in breast cancer cells. Stable clones expressing the hygromycn-resistant gene (HygB, control) or S194A mutant FADD (S194A FADD) were maintained as described in the ‘Materials and Methods’ and *in vitro* invasion activity was analysed by matrigel invasion assay.

**Figure 5:**
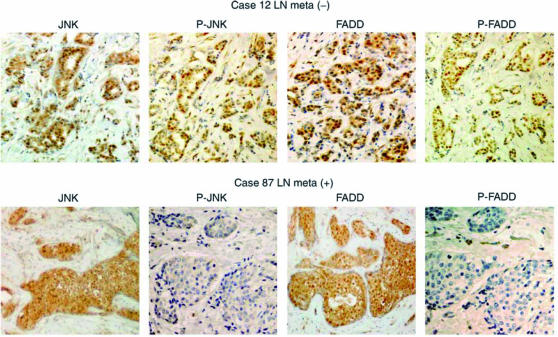
Expressions of phosphorylated JNK/FADD in breast cancer assessed by immunohistochemistry. Binding of antibodies to JNK and FADD and their phosphorylated forms were assessed with or without lymph node metastasis (Cases 87 and 12, respectively). P-JNK and P-FADD: phosphorylated forms of JNK and FADD.

**Figure 6:**
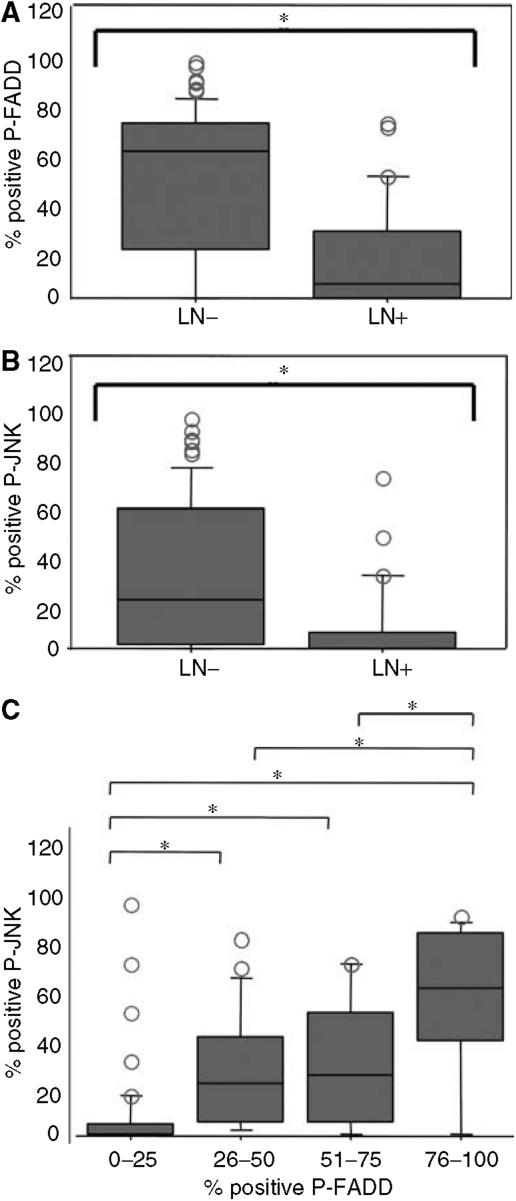
Correlations between P-JNK or P-FADD expression and lymph node metastasis, and between P-JNK and P-FADD expression were shown as box plots. (**A**, **B**) Expressions of P-JNK or P-FADD, in cases of lymph node metastasis positive or negative. (**C**) We classified 107 patients into four categories depending on the percentage of P-FADD expression immunohistochemically. The box plots showed the positivity of P-JNK in each of the four categories, and the correlation between P-JNK and P-FADD.

As shown in Figure 4, overexpression of a dephosphorylation mimicking mutant FADD, S194A FADD, enhanced invasion by both ER-positive (MCF-7) and -negative cells (MDA-MB-231) assessed by matrigel invasion assay. The result raises the possibility that FADD phosphorylation and upstream JNK activation can directly lead to the reduction of breast cancer metastasis. Immunohistochemical study revealed equal expression of JNK and FADD, but phosphorylated forms were significantly reduced in primary cancer cells with lymph node metastasis (Figure 5). Figure 6A and B show that each phosphorylated form of JNK and FADD was significantly highly expressed in the patients without lymph node metastasis. Furthermore, phosphorylated FADD was statistically correlated with JNK phosphorylation using *post hoc* test (Figure 6C) and Spearman's correlation test.

## DISCUSSION

We demonstrated here for the first time that long-term tamoxifen treatment causes G2/M cell cycle arrest through JNK/FADD phosphorylation in an ER-positive breast cancer cell line. In addition, the cytotoxic mechanisms mediated by tamoxifen appear to be executed by paclitaxel even in ER-negative cancer cells. We recently indicated an important role for JNK upstream of FADD phosphorylation at 194 serine on paclitaxel-induced apoptosis in human prostate cancer cells (Shimada *et al*, 2004). However, phosphorylated JNK/FADD signals alone cannot lead to apoptosis induction, but rather to cell cycle arrest at G2/M in breast cancer cells. Therefore, it seems likely that there are two major outcomes with phosphorylated FADD, apoptosis and cell cycle arrest. The phosphorylated FADD at 194 serine has been suggested to lead to G2/M transition, and casein kinase (CK) 1*α* specifically phosphorylates FADD at 194 serine and the phosphorylation of FADD is a crucial event for paclitaxel-induced cell cycle arrest in metaphase and cell proliferation (Alappat *et al*, 2003, 2005). It is also suggested that CK1 may provide a possible switch mechanism for Axin function in the regulation of Wnt and JNK pathways (Zhang *et al*, 2002), and demonstrated that mutant DN-Fas-associated death domain protected colon cancer cells from TRAIL-induced apoptosis in the presence of the CK1 inhibitor (Izeradjene *et al*, 2004). These studies indicated that CK1-induced downstream signals, JNK or FADD phosphorylation, are important to cell cycle regulation and apoptosis, which is in line with our study.

Long-term tamoxifen treatment has been reported to result in resistance, in which activation of EGF or the Her-2 receptor might be mainly involved (Knowlden *et al*, 2003). We also obtained several resistant clones after 0.01–0.1 *μ*M tamoxifen treatment for 4 months. However, neither P-JNK nor P-FADD were observed in such cells. Transfection with a phosphorylated mutant form of JNK or FADD successfully resulted in cell cycle arrest and growth suppression, similar to the data with higher (1 *μ*M) concentrations of tamoxifen (data not shown). Therefore, it is possible that activated JNK/FADD signals induced by paclitaxel can prevent relapse due to tamoxifen resistance. p53 stabilisation by long-term tamoxifen at 1 *μ*M was completely cancelled by JNK inhibitor or overexpression of S194A mutant FADD in the present study. In addition, we demonstrated p53 antisense oligonucleotide to inhibit growth arrest by tamoxifen to a similar extent. These results provide clear evidence that P-FADD plays an essential role in the p53 induction that contributes to cell cycle arrest at G2/M in breast cancer cells carrying wild-type p53. Of interest, in this context, the cytotoxic mechanism through activated JNK/FADD was executed by paclitaxel in an ER-negative cell line, MDA-MB-231 as well as in MCF-7 cells. This line has mutated p53, suggesting that signals other than p53 may be mainly involved downstream of JNK activation and p-FADD.

We found that Bcl-2 can be phosphorylated, dependent on phosphorylation of FADD at 194 serine, and Liao *et al* (2004) have reported a contribution of cyclin B1, CDC2 kinase complex and Bcl-2 phosphorylation to p53-independent cell cycle arrest with paclitaxel. We also observed inhibition of Bcl-2 phosphorylation when the dominant negative mutant form was overexpressed and cancelled cell cycle arrest with paclitaxel in MDA-MB-231 (data not shown). Therefore, in p53 mutated cancer cells, Bcl-2 phosphorylation might be a major downstream target of activated JNK/FADD signals. Thus, FADD phosphorylation at 194 serine is a common upstream participant of p53-dependent and -independent pathways of growth suppression mediated by tamoxifen or paclitaxel in human breast cancer cells.

Data on several prognostic factors have recently accumulated, including the hormone receptor status, Her-2 expression, lymph node metastasis and tumour size (Suo *et al*, 2002; Guerra *et al*, 2003; Cianfrocca and Goldstein, 2004), and overexpression of cyclins and Her-2 has been reported to enhance metastatic potential (Kuhling *et al*, 2003; Regitnig *et al*, 2004). Shimada *et al* (2005) demonstrated that phosphorylation status of FADD is associated with prostate cancer progression, using immunohistochemistry. Our present clinicopathological study demonstrated expression of phosphorylated forms of JNK and FADD to be significantly reduced in cancer cells with lymph node metastasis. Further, we provided evidence that overexpression of dephosphorylated FADD upregulates invasion activity of cancer cells *in vitro*. Since FADD is little phosphorylated at 194 serine in both MCF-7 and MDA-MB-231 cells without any treatment, reduced expression of phosphorylated FADD by S194A mutant FADD may be insignificant. In addition, overexpression of mutant FADD mimicking phosphorylated form (S194E or S194D) made no significant effects on invasion activity in these cells (data not shown). Therefore, we consider that upregulation of dephosphorylated FADD rather than reduction of phosphorylated FADD contributes to enhancement of invasion activity in breast cancer cells.

**Figure 7:**
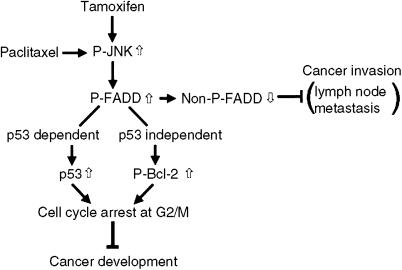
Schematic presentation of JNK/FADD-mediated regulation of cytotoxicity. Tamoxifen or paclitaxel activates JNK and induces FADD phosphorylation, resulting in cell cycle arrest and suppression of cancer growth through p53 stabilisation or Bcl-2 phosphorylation in breast cancer cells. In addition, the nonphosphorylated form of FADD is closely associated with cancer invasion, therefore, induction of FADD phosphotrylation can prevent metastatic activity.

We examined pathological correlations between FADD phosphorylation at 194 serine and ER or p53 status, but the results were insignificant. However, the present *in vitro* analysis demonstrated that JNK/FADD signals affect not only in ER-positive cancer cells, MCF-7, but also in ER-negative cells, MDA-MB-231. In addition, as shown in Figure 7, Bcl-2 phosphorylation other than p53 plays an important role in JNK/FADD signals-mediated growth arrest of breast cancer. Therefore, we conclude that JNK/FADD signals and the related growth regulation can be executed even in ER-negative cancer cells with mutated p53.

Taking the available data together, phosphorylation of FADD via JNK activation may lead not only to cell growth suppression through G2/M arrest but also directly to inhibition of cancer invasion or lymph node metastasis. As lymph node metastasis is well known to be strongly associated with prognosis of breast cancers (Colpaert *et al*, 2001), assessment of the phosphorylation status of JNK or FADD might provide relevant information for the efficacy of clinical therapy. In other words, drugs that can activate JNK/FADD such as paclitaxel might be best indicated for the cases with low levels of phosphorylated JNK or FADD expression. This is in line with the results of a previous clinical study on the outcome of paclitaxel treatment in lymph node-positive primary breast cancers (Fumoleau *et al*, 2003; Henderson *et al*, 2003).

In summary, the present study indicates for the first time that activation of the JNK/FADD pathway might be very important for suppression of cell growth and invasion, especially lymph node metastasis, in both ER-positive and -negative breast cancers. We conclude that chemotherapy targeting to the JNK/FADD signalling warrants further attention.

## Figures and Tables

**Table 1:** Clinicopathological characteristics of 107 patients

**Characteristics**	**No. of patients (*n*=107) (%)**
*Age*	
<50	34 (31.8)
⩾50	73 (68.2)
	
*Tumour size (cm)*	
<2	37 (34.6)
⩾2	70 (65.4)
	
*Lymph node status*	
Positive	36 (33.6)
Negative	71 (66.4)
	
*Oestrogen receptor*	76 (71.0)
Positive	31 (29.0)
Negative	
	
*Progesterone receptor*	
Positive	55 (51.4)
Negative	52 (48.6)
	
*c-erbB-2 receptor*	
Positive	29 (27.1)
Negative	78 (72.9)
